# Prediction model of early biomarkers of massive cerebral infarction caused by anterior circulation occlusion: Establishment and evaluation

**DOI:** 10.3389/fneur.2022.903730

**Published:** 2022-08-18

**Authors:** Jingshu Chen, Jinze Li, Zhihua Xu, Luojin Zhang, Shouliang Qi, Benqiang Yang, Zimeng Chen, Xinrui Wang, Yang Duan

**Affiliations:** ^1^Center for Neuroimaging, Department of Radiology, General Hospital of Northern Theater Command, Shenyang, China; ^2^Center for Neuroimaging, Northern Theater Command Postgraduate Training Base of Jinzhou Medical University General Hospital, Shenyang, China; ^3^Department of Radiology, Tong De Hospital of Zhejiang Province, Hangzhou, China; ^4^Center for Neuroimaging, Northern Theater Command Postgraduate Training Base of Dalian Medical University General Hospital, Shenyang, China; ^5^College of Medicine and Biological Information Engineering, Northeastern University, Shenyang, China; ^6^Department of Radiology, General Hospital of Northern Theater Command, Shenyang, China; ^7^Boston University College of Art and Science, Boston, MA, United States

**Keywords:** massive cerebral infarction, anterior circulation occlusion, prediction model, early imaging signs, CT

## Abstract

**Objective:**

The purpose of this study is to establish and evaluate an early biomarker prediction model of massive cerebral infarction caused by anterior circulation occlusion.

**Methods:**

One hundred thirty-four patients with acute cerebral infarction from January 2018 to October 2020 were selected to establish the development cohort for the internal test of the nomogram. Ninety-one patients with acute cerebral infarction hospitalized in our hospital from December 2020 to December 2021 were constituted the validation cohort for the external validation. All patients underwent baseline computed tomography (CT) scans within 12 h of onset and early imaging signs (hyperdense middle cerebral artery sign, obscuration of the lentiform nucleus, insular ribbon sign) of acute cerebral infarction were identified on CT by two neurologists. Based on follow-up CT images, patients were then divided into a massive cerebral infarction group and a non-massive cerebral infarction group. The nomogram model was constructed based on logistic regression analysis with R language. The nomogram was subsequently validated in an independent external validation cohort. Accuracy and discrimination of the prediction model were evaluated by a calibration chart, receiver operating characteristic (ROC) curve, and decision curve.

**Results:**

The indicators, including insular ribbon sign, reperfusion therapy, National Institutes of Health Stroke Scale (NHISS) score, previous cerebral infarction, and atrial fibrillation, were entered into the prediction model through binary logistic regression analysis. The prediction model showed good predictive ability. The area under the ROC curve of the prediction model was 0.848. The specificity, sensitivity, and Youden index were 0.864, 0.733, and 0.597, respectively. This nomogram to the validation cohort also showed good discrimination (AUC = 0.940, 95% CI 0.894–0.985) and calibration.

**Conclusion:**

Demonstrating favorable predictive efficacy and reproducibility, this study successfully established a prediction model of CT imaging signs and clinical data as early biomarkers of massive cerebral infarction caused by anterior circulation occlusion.

## Introduction

Massive cerebral infarction (MCI) occurs in approximately 10.0% of all strokes and has a mortality rate of up to 80.0% (1, 2). MCI is a severe ischemic stroke caused by occlusion of the proximal middle cerebral artery (MCA) or internal carotid artery terminus (ICA). Relative studies have reported on the benefits in clinical practice of early imaging signs of acute cerebral infarction on computed tomography (CT) (3–5). Therefore, it is vital to establish a prediction model based on early clinical and imaging data to facilitate the early diagnosis of MCI caused by anterior circulation occlusion. A prediction model can provide an effective time window and outcome prediction of endovascular therapy in MCI patients.

## Materials and methods

### Population

Acute cerebral infarction patients with clinical and imaging data were enrolled in this study according to the following inclusion criteria: (1) hospital admission within 12 h after onset of signs/symptoms; (2) infarction lesions that could not be seen on their first non-contrast CT scan (performed on admission prior to treatment); (3) patients with clinical symptoms who had unilateral ICA or MCA occlusion confirmed by CT angiography (CTA); (4) head CTA and routine CT exams completed within 12 h of onset; and (5) follow-up CT that was performed within 24 h after stroke onset showing cerebral infarction. The exclusion criteria were as follows: (1) patients with malignancy or aneurysm; (2) baseline CT that showed massive cerebral and hemorrhagic infarction; (3) vascular occlusion after cranial surgery; (4) allergy to contrast media; and (5) patients with posterior circulation infarction.

### Image acquisition and analysis

Computed tomography scans were obtained for all patients included in the study with the GE Discovery CT 750 HD scanner, USA (GE Healthcare, Milwaukee, USA) using the following parameters: 120 kV tube voltage, 264 mAs tube current, slice thickness of 5 mm, field of view (FOV) of 240 × 240, and matrix of 512 × 512.

**Figure 1:**
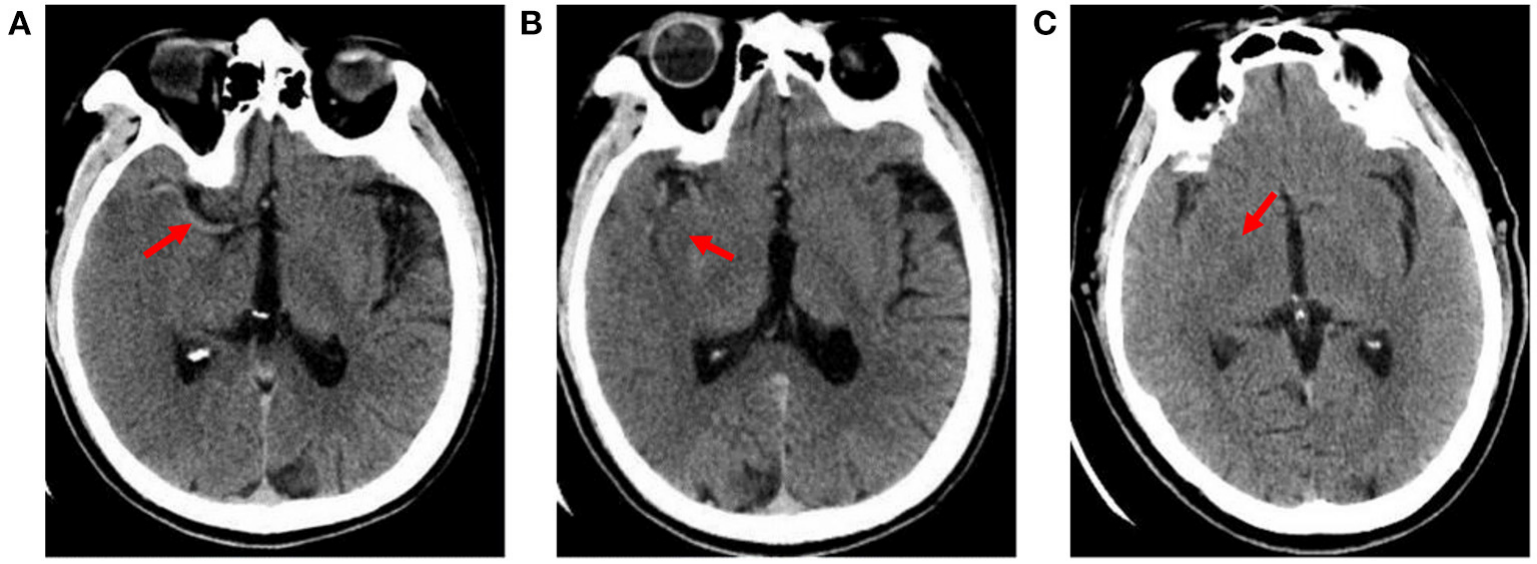
Early imaging signs on CT. **(A)** hyperdense middle cerebral artery sign: the M1 segment of the right middle cerebral artery shows high density (X-ray uptake value of 64 HU); **(B)** insular ribbon sign: the gray-white matter interface of the right insular zone disappears, and the cerebral sulcus becomes shallow; **(C)** obscuration of the lentiform nucleus: the right lentiform nucleus structure is blurred, and the density is reduced (the CT average value is 27.4 HU; the CT average value of the same area on the healthy side is 34 HU).

The hyperdense middle cerebral artery sign (HMCAS) can be defined qualitatively as any artery that appears denser than adjacent or equivalent contralateral arteries (6, 7). However, quantitative definitions [>43 Hounsfield units (HU) or >1.2-fold the density of a normal contralateral vessel] have also been proposed (8, 9). Other signs include assessment of the difference in density of obscuration of the lentiform nucleus and insular ribbon sign between the affected and healthy side (10). All image variables were measured independently by two neuroradiologists with extensive experience who were blinded to symptoms or side effects. Cases of disagreement between two observers were settled by consensus (Figure 1).

MCI was defined as infarction area ≥20 cm^2^ or lesions involving more than two lobes (11, 12).

### Statistical analysis

Statistical analysis of the data was performed using R4.1.2 software (The R Foundation for Statistical Computing, Vienna, Austria) and SPSS v.22.0 (SPSS Statistics, IBM Corporation, Armonk, New York). Frequencies or percentages expressed categorical variables (Chi-square tests). Normally distributed continuous variables: the mean ± standard deviation (independent sample *t*-tests). Non-normally distributed data: the median and interquartile range (IQR; the Man–Whitney *U*-test). All predictive factors were analyzed using binary logistic regression. A nomogram model that can predict the risk of MCI was built by selecting the best predictive factors. The validity of this nomogram model was checked on an external validation cohort considering calibration. The receiver operating characteristic (ROC) curve was used to quantify the model resolution ability and further Bootstrap verified on the nomogram. Decision-curve analysis was conducted in the validation cohort. Net benefit at different threshold probabilities in patient information was quantified. The decision curve was used to assess the clinical utility and operability of the nomogram. The degree of interobserver agreement in the subjective evaluation of early imaging signs was evaluated by cross-tabulation and calculation of kappa (κ values). A kappa of one indicated complete agreement of all observations in all cases, whereas a kappa of zero indicated that any observed agreement was attributed to chance.

## Results

### Cohort description

#### Development cohort

Over the 134 study patients in the development cohort, 92 (68.7%) were males, with a mean age of 63.9 years (range, 31.0–92.0). Of these, 75 (56.0%) patients occurred with MCI. Compared to the non-MCI group, the MCI group had higher NIHSS (median 15.0 vs. 13.0). And the MCI group was more likely to show early signs (HMCAS, obscuration of the lentiform nucleus, insular ribbon sign). The differences between patients with MCI and non-MCI group in atrial fibrillation (*P* = 0.040), reperfusion therapy (*P* = 0.012), obscuration of the lentiform nucleus (*P* = 0.000), insular ribbon sign (*P* = 0.000), and the early joint signs were significant (Table 1). The interclass correlation analysis between the two observers showed excellent agreement for HMCAS (κ value 0.640), obscuration of the lentiform nucleus (κ value 0.741), and insular ribbon sign (κ value 0.821).

**Table 1:** General characteristics for development and validation cohort.

**Characteristics**	**Development cohort**				**Validation cohort**			
	**ALL**	**MCI**	**Non-MCI**	***P*-value**	**ALL**	**MCI**	**Non-MCI**	***P*-value**
	**(*n* = 134)**	**(*n* = 75)**	**(*n* = 59)**		**(*n* = 91)**	**(*n* = 51)**	**(*n* = 40)**	
Gender: male, *n* (%)	92/42	48/27	44/15	0.192	72/19	39/12	33/7	0.485
Age: years	63.9 ± 12.4	64.8 ± 12.4	62.9 ± 12.3	0.959	64.7 ± 10.8	64.0 ± 12.0	65.5 ± 9.1	0.053
Admission NIHSS	14.0 (8.0–17.0)	15.0 (11.0–18.0)	13.0 (8.0–17.0)	0.058	12.0 (4.0–17.0)	14.0 (11.0–19.0)	3.5 (1.0–9.8)	0.000
Systolic blood pressure on admission (mmHg)	151.0 (133.0–168.0)	150.0 (133.0–171.0)	152.0 (136.0–165.0)	0.966	154.2 ± 24.8	157.8 ± 26.7	149.6 ± 21.6	0.667
Diastolic blood pressure on admission (mmHg)	88.0 (75.8–99.0)	87.0 (75.0–99.0)	89.0 (77.0–99.0)	0.950	87.0 (80.0–96.0)	88.0 (81.0–98.0)	85.5 (80.0–91.8)	0.149
Atrial fibrillation: *n* (%)	42 (31.3)	29 (38.7)	13 (22.0)	0.040	19 (20.9)	15 (29.4)	4 (10.0)	0.025
Hypertension: *n* (%)	79 (59.0)	47 (62.7)	32 (54.2)	0.327	64 (70.3)	35 (68.6)	29 (72.5)	0.690
Diabetes: *n* (%)	34 (25.4)	15 (20.0)	19 (32.2)	0.108	27 (29.7)	12 (23.5)	15 (37.5)	0.150
Smoking: *n* (%)	76 (56.7)	42 (56.0)	34 (57.6)	0.851	44 (48.4)	25 (49.0)	19 (47.5)	0.886
Alcohol drinking: *n* (%)	70 (52.2)	38 (50.7)	32 (54.2)	0.682	41 (45.1)	22 (43.1)	19 (47.5)	0.680
Coronary heart disease: *n* (%)	26 (19.4)	15 (20.0)	11 (18.6)	0.844	6 (6.6)	3 (5.9)	3 (7.5)	0.759
Previous cerebral infarction: *n* (%)	24 (17.9)	10 (13.3)	14 (23.7)	0.121	22 (24.2)	7 (13.7)	15 (37.5)	0.009
Hypercholesterolemia: *n* (%)	58 (43.3)	29 (38.7)	29 (49.2)	0.226	48 (52.7)	28 (54.9)	20 (50.0)	0.644
Reperfusion therapy: *n* (%)	94 (70.1)	46 (61.3)	48 (81.4)	0.012	29 (31.9)	21 (41.2)	8 (20.0)	0.032
HMCAS: *n* (%)	55 (41.0)	32 (42.7)	23 (39.0)	0.668	48 (52.7)	30 (58.8)	18 (45.0)	0.192
Obscuration of the lentiform nucleus: *n* (%)	62 (46.3)	45 (60.0)	17 (28.8)	0.000	12 (13.2)	11 (21.6)	1 (2.5)	0.008
Insular ribbon sign: *n* (%)	67 (50.0)	54 (72.0)	13 (22.0)	0.000	33 (36.3)	31 (60.8)	2 (5.0)	0.000
HMCAS + obscuration of the lentiform nucleus: *n* (%)	27 (20.1)	20 (26.7)	7 (11.9)	0.035	8 (8.8)	7 (13.7)	1 (2.5)	0.062
HMCAS +insular ribbon sign: *n* (%)	26 (19.4)	22 (29.3)	4 (6.8)	0.001	21 (23.1)	19 (37.3)	2 (5.0)	0.000
Obscuration of the lentiform nucleus + insular ribbon sign: *n* (%)	46 (34.3)	39 (52.0)	7 (11.9)	0.000	9 (9.9)	9 (17.6)	0 (0.0)	0.005
HMCAS + obscuration of the lentiform nucleus + insular ribbon sign: *n* (%)	20 (14.9)	17 (22.7)	3 (5.1)	0.005	6 (6.6)	6 (11.8)	0 (0.0)	0.026
TC (mmol/L)	4.4 ± 1.2	4.6 ± 1.2	4.2 ± 1.1	0.354	4.2 ± 1.1	4.3 ± 1.2	4.1 ± 1.0	0.198
TG (mmol/L)	1.3 (0.9–1.9)	1.3 (0.9–1.8)	1.4 (0.9–1.9)	0.865	1.2 (0.9–1.5)	1.0 (0.8–1.5)	1.3 (1.1–1.6)	0.068
HDL-C (mmol/L)	1.2 (0.9–1.4)	1.2 (1.0–1.4)	1.2 (0.9–1.4)	0.665	1.2 (1.1–1.4)	1.2 (1.1–1.4)	1.2 (1.0–1.3)	0.701
LDL-C (mmol/L)	2.4 (1.8–3.0)	2.5 (2.0–3.4)	2.2 (1.7–2.9)	0.078	2.5 ± 0.9	2.5 ± 0.9	2.4 ± 0.8	0.248
LPa (mmol/L)	192.4 (75.9–383.6)	158.3 (71.3–314.3)	210.0 (83.5–394.3)	0.297	153.3 (68.0–342.2)	194.1 (84.8–364.3)	111.9 (58.6–284.0)	0.104
PLT (10^9^/L)	208.5 (173.3–262.5)	214.0 (179.0–285.0)	204.0 (162.0–242.0)	0.126	232.5 ± 74.8	228.0 ± 77.1	238.3 ± 72.4	0.467
PT (s)	13.8 (13.2–14.4)	13.8 (13.3–14.4)	13.6 (13.1–14.3)	0.526	13.4 (12.9–14.3)	13.7 (13.1–14.4)	13.3 (12.8–13.9)	0.152
TT (s)	16.5 (15.7–17.6)	16.5 (15.8–17.6)	16.5 (15.6–17.7)	0.905	16.4 (15.7–17.9)	16.6 (15.9–18.1)	16.1 (15.5–17.3)	0.174
APTT (s)	36.9 (33.6–40.4)	36.2 (33.4–40.0)	37.5 (34.1–41.4)	0.255	35.8 (33.4–40.9)	36.0 (33.5–41.5)	35.1 (33.2–39.6)	0.443
FIB (g/L)	4.4 (3.0–5.8)	4.5 (3.2–5.8)	4.4 (3.0–5.8)	0.722	3.8 (3.2–5.1)	3.8 (3.3–5.1)	3.9 (3.1–5.0)	0.686

NIHSS, National Institutes of Health Stroke Scale; HMCAS, hyperdense middle cerebral artery sign; TC, total cholesterol; TG, triglyceride; HDL-C, high-density lipoprotein cholesterol; LDL-C, low-density lipoprotein cholesterol; LPa, lipoprotein a; PLT, platelet; PT, prothrombin time; TT, thrombin time; APTT, activated partial thromboplastin time; FIB: fibrinogen.

#### Validation cohort

A total of 91 patients (mean age, 64.7 ± 10.8 years), of which 51 (56.0%) occurred MCI, were included in the validation cohort. The differences between patients with MCI group and non-MCI group in NIHSS (*P* = 0.000), atrial fibrillation (*P* = 0.025), previous cerebral infarction (*P* = 0.009), reperfusion therapy (*P* = 0.032), obscuration of the lentiform nucleus (*P* = 0.008), insular ribbon sign (*P* = 0.000), and the early joint signs were significant (Table 1).

### Predictors selection

Variables with *P* < 0.2 in the univariate analysis were considered in the multivariate model. Multivariate logistic regression analysis used by a backward stepwise method was conducted to determine the optimal features in the development cohort to establish a predictive nomogram. The five optimal features included insular ribbon sign, reperfusion therapy, NHISS, previous cerebral infarction, and atrial fibrillation (Table 2).

**Table 2:** Results of each factor analysis in the logistic regression model of MCI.

**Influencing factors**	**β**	**SE**	***P***	**OR (95%CI)**
Insular ribbon sign	2.314	0.464	0.006	10.114 (4.246–26.489)
Reperfusion therapy	−2.287	0.663	0.000	0.102 (0.025–0.343)
NHISS	0.084	0.039	0.003	1.088 (1.011–1.178)
Previous cerebral infarction	−1.446	0.618	0.002	0.235 (0.066–0.759)
Atrial fibrillation	1.066	0.517	0.004	2.903 (1.075–8.294)

CI, confidence interval; NIHSS, National Institutes of Health Stroke Scale; OR, Odds ratio.

### Nomogram model

**Figure 2:**
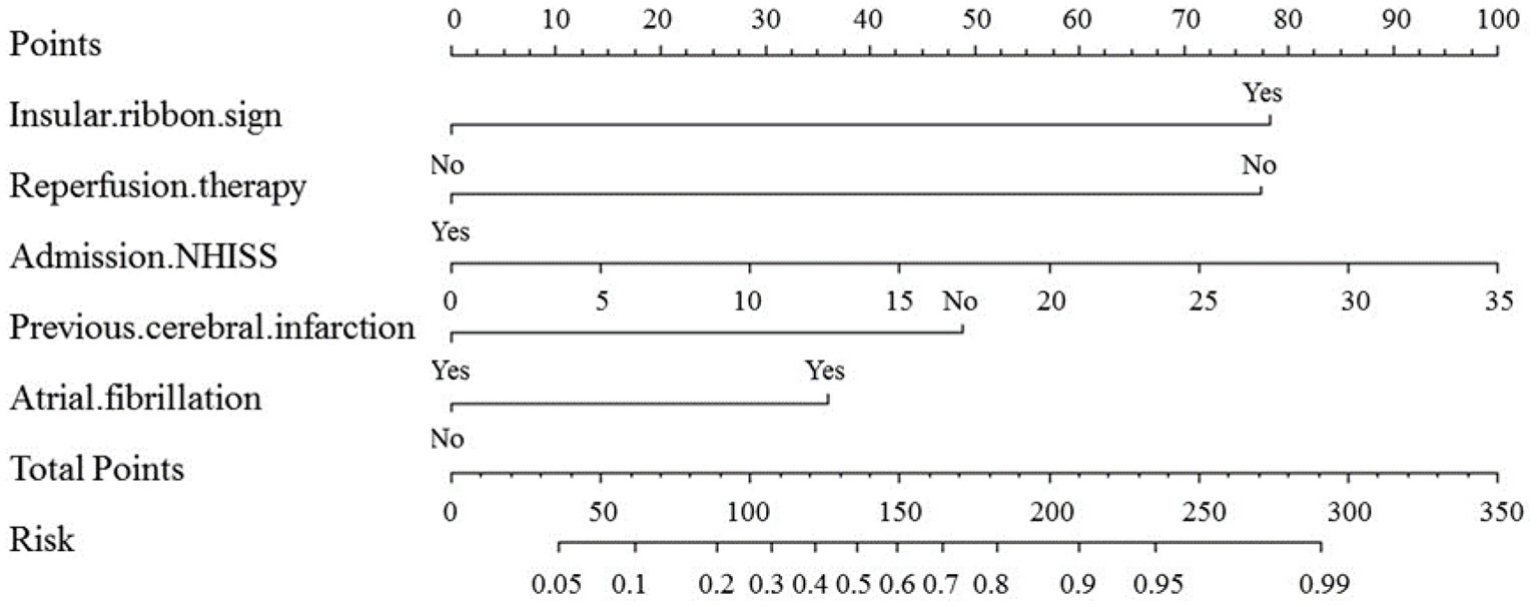
Nomogram for predicting the probability of MCI based on insular ribbon sign, reperfusion therapy, NHISS, previous cerebral infarction and atrial fibrillation. Nomogram calculates the occurrence of MCI by assigning each predictive variable with scales in line segments and integrating the scores.

Variables in the multivariate logistic regression in the development cohort were the same as those in the validation cohort. The above five optimal predictors were incorporated into nomograms using the R software (Figure 2). The total score based on the values of the predictive variables was calculated, and then the risk of MCI was calculated based on the total score.

### Test of the nomogram prediction model

**Figure 3:**
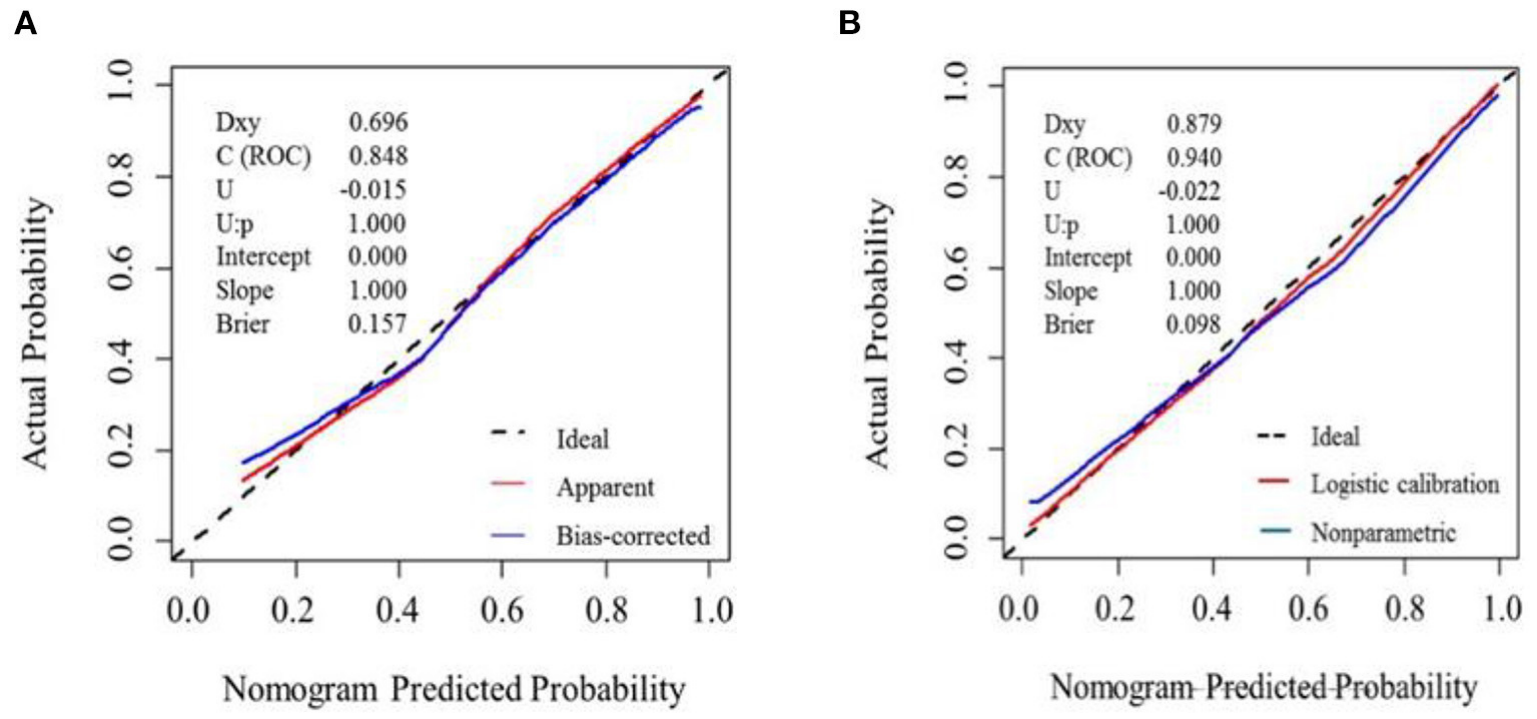
Calibration plot for nomogram in the **(A)** development cohort and **(B)** validation cohort. The *x*-axis represents the predicted probability and *y*-axis represents the actual probability. A subset of various statistics useful for validating the model are also shown. Dxy: Somers' Dxy rank correlation between p (predicted possibilities) and y (actual outcome = 0 or 1). C (ROC): the ROC area. U, Unreliability index. Brier: average squared difference in p and y. **(A)** Dashed line (“Ideal”) represents ideal predictions. The red line represents the entire cohort (*n* = 134), and the blue line indicats observed nomogram performance by bias-corrected by bootstrapping (B = 1,000). **(B)** Dashed line denotes perfect calibration. A smoothing curve (blue) and the calibration curve (red) basically coincide.

The nomogram model correction curve was calibrated against the consistency between the predicted risks and actual observed results. The diagonal dashed line indicates the perfect prediction of the ideal model, and the closer the diagonal dashed line, the better the prediction. The ability to predict MCI in the validation cohort and development cohort was ideal, as Figure 3 displayed.

**Figure 4:**
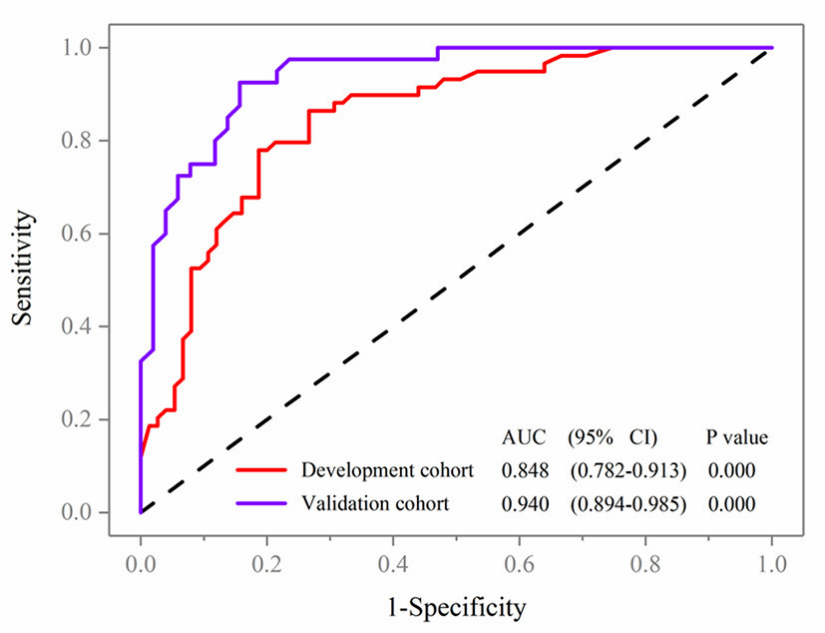
Receiver operating characteristic curve analyses of prediction for MCI in the development and validation cohort.

Variables in the multivariate logistic regression in the development cohort were the same as those in the validation cohort. The ROC analyses of predicting MCI in the development and validation cohort were demonstrated in Figure 4. With AUCs (95% CI) of 0.848 (0.782–0.913) and 0.940 (0.894–0.985), significant difference was found between the development and validation cohorts (DeLong test: *P* = 0.025; Figure 4). Compared to the development cohort, the validation cohort with higher accuracy (85.7 vs. 79.1%), sensitivity (84.3 vs. 73.3%), specificity (92.5 vs. 86.4%), negative predictive value (NPV; 87.5 vs. 79.7%) and positive predictive value (PPV; 84.3 vs. 78.7%).

**Figure 5:**
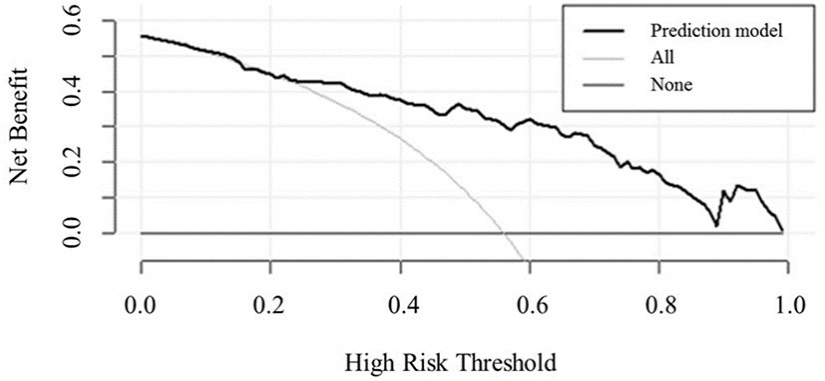
Decision curve analysis comparing the clinical usefulness of MCI. The clinical usefulness is the net benefit (*y*-axis) of using the score to risk stratify patients relative to two extreme strategies of treating all the patients and treating none of the patients across a range of prespecified threshold probabilities (*x*-axis).

**Figure 6:**
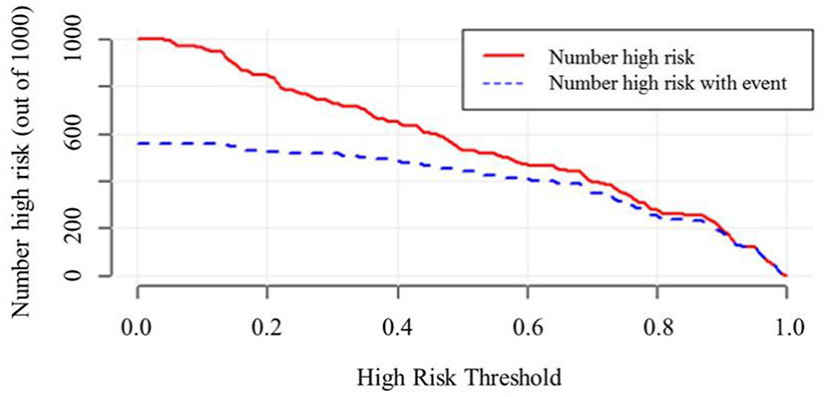
Clinical impact curve of model nomogram. The red curve (Number high risk) indicates the number of people who are. The blue curve (Number of high risk with outcome) indicates the number of true positives. When the threshold probability was >90.0% of the predicted probability value, the prediction model determined that the MCI high-risk population was highly matched with the actual MCI population, confirming the high clinical efficiency of the prediction model.

The decision curve (Figure 5) analysis showed that clinical decisions based on the prediction model were beneficial (in the threshold probability range over 20.0%) and implied the practical clinical application and operability of the prediction model. Clinical impact curve analysis was used to predict the risk stratification for every 1,000 people. When the threshold probability was over 90.0%, the event occurrence probability was utterly consistent with the model, suggesting that the model had good prediction performance (Figure 6).

## Discussion

A nomogram is a graphical representation of a clinical prediction model built on multivariate regression analysis, performed by assigning scores based on predictive variable values, then calculating total scores and converting them to estimate outcome risk. The results of a prediction model have higher readability and practical value. Thus far, there have been few studies on prediction model based on early CT imaging signs and clinical risk factors of MCI. Hence, this study focuses on establishing a prediction model of early CT imaging signs and clinical information in order to help clinicians identify patients with MCI earlier, give timely targeted treatment, and maximize the quality of life and prognosis of patients.

This study demonstrates that the insular ribbon sign can be used as an independent predictor of MCI. The principle of the insular ribbon sign is that the cerebral tissue in the ischemic area begins to become edematous after cerebrovascular occlusion due to narrowing or occlusion of the perforator arteries that supply the insular region. Since perforator arteries are first affected by hemodynamic alterations, ischemia and infarction occur earliest in the insular region. Within 3–6 h after the onset of infarction, CT early low-density changes (insular ribbon signs, obscuration of the lentiform nucleus) exceeded 50.0% of the area supplied by the MCA, with 61.0% sensitivity and 94.0% specificity in predicting massive infarction. The earlier the CT examination is performed, the higher specificity and the lower sensitivity of its findings (13–16). In addition, obscuration of the lentiform nucleus appears within 4–6 h, and the HMCAS appears within 30 min of onset; these findings have a specificity of 85.0%−100.0% (12, 17, 18).

In this study, results of logistic regression analysis showed that the HMCAS was not an independent predictor of MCI, though some relevant studies have shown a close relationship between the HMCAS and the dependent variable. After multiple CT, CTA, MRI, MRA, and autopsy examinations, the study confirmed that the HMCAS had low sensitivity (only 7.0%−69.0%) and high specificity (85.0%−100.0%) in the diagnosis of cerebral infarction (12, 19–21). Cerebral infarction may be diagnosed if the HMCAS is present (14, 22). The appearance of the HMCAS is a transient phenomenon, and its presence may be related to the hypercoagulable state of blood or the formation of emboli. Once the thrombus dissolves or its density gradually changes, the HMCAS will disappear, so the positive rate of the HMCAS is high in the early stage of disease (20, 23). Theoretically, the HMCAS should be present at the early stage of an occlusion that occurs in the MCA area. However, Leys et al. (20) and Manelfe et al. (14) reported that the occurrence rate of HMCAS is low, 30.9 and 17.7% respectively, which is consistent with our results. There are many reasons that contribute to the low occurrence rate of the HMCAS and mainly include the following: (1) the time from onset to the first CT examination in patients with cerebral infarction is different. Numerous studies have shown that the HMCAS detection rate gradually decreases within 3, 6, 12–24 h, and 6–8 days after the onset of the disease (13, 21, 24, 25). Some patients have been confirmed to have MCA infarction by CTA, but the HMCAS does not appear because those patients have not only MCA occlusion but also ICA occlusion, resulting in an extension of the thrombus. Therefore, the HMCAS may not appear immediately after onset. Based on this, CT scans should be performed earlier to help improve the detection rate of the HMCAS; (2) in the ultra-early stage of cerebral infarction, the HMCAS is not obvious, so it is difficult to correctly detect the HMCAS. When the radiologist knows that the patient has a clinical presentation of MCA or detects the HMCAS in one of two CT examination comparisons, the positive rate of the HMCAS tends to increase (18).

The results from clinical data of this study showed that the NIHSS score, reperfusion therapy, previous cerebral infarction, and atrial fibrillation are the best predictors of the occurrence of MCI. The NHISS score is highly comprehensive and has been widely used in clinical practice to assess stroke, and a high NIHSS score is now generally accepted in predicting MCI outcome (26). However, some studies (27) have found that an NIHSS score ≥15 in patients with right hemisphere infarction or an NIHSS score ≥20 in patients with left hemisphere infarction is a risk factor for massive occupancy effects. The NIHSS score emphasizes aphasia and therefore may be overestimated in the evaluation of patients with dominant hemispheric infarction. Timely reperfusion therapy in patients with acute ischemic cerebral infarction can revascularize reversibly damaged cerebral tissue around the infarction, or the “ischemic penumbra,” which can reduce the final infarct volume of stroke. The earlier the treatment is performed, the better the prognosis. The speed of development of the ischemic penumbra is mainly influenced by collateral circulation. Timely establishment of collateral circulation is conducive to restore the blood supply of the ischemic penumbra and greatly improve the blood circulation status. In this study, 61.3% of patients in the MCI group were treated with reperfusion, and poor collateral circulation may be the reason for this phenomenon. Cerebral cells are permanent and non-renewable. Patients with previous cerebral infarction do not have a significantly increased risk of secondary cerebral infarction. In this study, only 13.3% of patients in the MCI group had a secondary cerebral infarction; this may be related to timely medication, active control of hypertension, hyperlipidemia, hyperglycemia, and habits (no smoking, no alcohol, and doing exercise). Atrial fibrillation is one of the important risk factors for cerebral infarction. Once the mural thrombus formed by atrial fibrillation detaches and enters the internal carotid artery system, it will lead to sudden interruption of blood flow in the cerebral tissue, and collateral circulation cannot be established in time. Therefore, the lesion infarction area is large, and the disease progress is rapid.

In addition, studies have shown that nausea and vomiting occurring within 24 h of disease onset, increased body temperature, peripheral leukocytes, infarct size ≥MCA blood supply range within 48 h, the Alberta Stroke Program Early CT Score (ASPECTS), early MRI score, and collateral circulation score within 8 h of onset can be used as early predictors of MCI. Among them, infarct size ≥MCA blood supply range and increased body temperature have relatively higher accuracy in predicting MCI (28, 29). Previous studies (30, 31) have demonstrated that aging, hypertension, smoking, alcohol drinking, systolic blood pressure, and diastolic blood pressure at admission are risk factors for the development of MCI. However, these results were not obtained in this study, probably due to the single-center and small sample size. Multicenter and large sample size studies are required in the future.

There are several limitations of this study. First, this study is a retrospective study with small sample size. Second, CT was the only imaging modality. Finally, CT imaging with a 5 mm thickness and a 3 mm reconstruction may result in a reduced positive rate of the HMCAS, obscuration of the lentiform nucleus and insular ribbon sign.

In conclusion, based on early imaging signs and clinical data, this study provided a good prediction model to improve the sensitivity, specificity, and accuracy of early prediction of MCI. In the meantime, it will help clinicians improve patient outcomes and reduce the mortality and disability rate of MCI.

## Data availability statement

The raw data supporting the conclusions of this article will be made available by the authors, without undue reservation.

## Ethics statement

The studies involving human participants were reviewed and approved by the Ethics Committee of the General Hospital of Northern Theater Command. The patients/participants provided their written informed consent to participate in this study.

## Author contributions

YD and JC contributed to the idea and manuscript preparation. BY, SQ, XW, and ZC helped perform the analysis with constructive discussions and revise manuscript. JL and LZ collected the imaging data. All authors discussed the results and revised the manuscript.

## Funding

Funding for this study was provided in part by Project of Natural Science Foundation of Shenyang (grant no. 20-205-4-044), Project of Natural Science Foundation of Liaoning Province (grant no. 201602768), and Project of Scientific Research Foundation for the PhD of Liaoning Province (grant no. 2019-BS-267).

## Conflict of interest

The authors declare that the research was conducted in the absence of any commercial or financial relationships that could be construed as a potential conflict of interest.

## Publisher's note

All claims expressed in this article are solely those of the authors and do not necessarily represent those of their affiliated organizations, or those of the publisher, the editors and the reviewers. Any product that may be evaluated in this article, or claim that may be made by its manufacturer, is not guaranteed or endorsed by the publisher.
